# Impact of Foliar Fertilization on Growth, Flowering, and Corms Production of Five *Gladiolus* Varieties

**DOI:** 10.3390/plants10091963

**Published:** 2021-09-20

**Authors:** Endre Kentelky, Zsolt Szekely-Varga

**Affiliations:** Department of Horticulture, Faculty of Technical and Human Sciences, Sapientia Hungarian University of Transylvania, Sighișoarei 1/C, 540485 Targu Mures, Romania; szekelyvarga.zsolt@gmail.com

**Keywords:** corms, foliar fertilization, *Gladiolus*, vase durability

## Abstract

Degraded and salt affected soils are appearing more often in cultivated areas. These specific problems could reduce nutrient uptake, which can result in quality and yield loss of the cultivated plants. In order to cope with this pedo-climatic condition growers are applying fertilizers; however, due to inadequate application, soil degradation will continue. Five *Gladiolus* varieties were subjected to foliar fertilization treatments to assess the effect on the plant’s growth parameters, vase durability and daughter corm production. Our results indicate that plants treated with foliar fertilization show significant increase in the measured parameters, flower stem length, vase durability and daughter corm production. In conclusion, our study suggests that application of foliar fertilization can increase *Gladiolus* plants decoration and propagation, even with a smaller footprint on nature.

## 1. Introduction

Currently in the world 20% of the cultivated land is degraded and salt affected, which is affecting nutrient uptake and resulting in quality and yield reduction of the cultivated plants. More importantly, these factors are contributing to crop losses worldwide [1,2]. According to several studies, in order to cope with these conditions and increase production, chemical fertilizers are applied, but due to inappropriate application, soil degradation (acidification; salinization; nutrient imbalance; and irregular accumulation of nitrogen, phosphorus and potassium) occurs in the cultivated lands [3,4].

Fertilization can be divided into two main methods: root and foliar fertilization [4]. Foliar fertilization can be absorbed directly through the leaves and can be transported more quickly and efficiently to the other plant organs compared to root fertilization [5,6]. Moreover, foliar fertilization can be sprayed at optimum times and concentrations, according to the requirement of different plants, at different growth stages. This type of fertilization can be more suitable to the plant’s needs, in contrast to root fertilization [4,7,8].

*Gladiolus* genus is a perennial, monocotyledonous, geophyte, semi-rustic ornamental plant and includes about 260 species [8,9]. The *Gladiolus* originates in Mediterranean Europe, Asia, and South and Tropical Africa [10,11,12,13]. It can be found as an ornamental garden plant and also as cut flowers used for bouquets and arrangements [13]. These majestic plants are found in different shapes, colours, and sizes, and can be cultivated almost everywhere, but should be considered for regions where spring and summer conditions are favourable [14].

The main aim of the present research was to test the responses to foliar fertilization of five highly cultivated *Gladiolus* varieties. ‘Black Beauty’, ‘Green Star’, ‘Nova Lux’, ‘Zizane’, and ‘Frizzled Coral Lace’ were analysed in the study. The effect of three foliar fertilizers—Fitofolis, Bionat Plus and Cropmax—and the mixture of the three on the flower quality and the amount of new daughter corms produced by the selected gladioli was investigated. We expected to establish whether any of the five varieties was more suitable for cultivation in the climate conditions of the Carpathian Basin.

## 2. Results

### 2.1. Plant Growth

**Figure 1:**
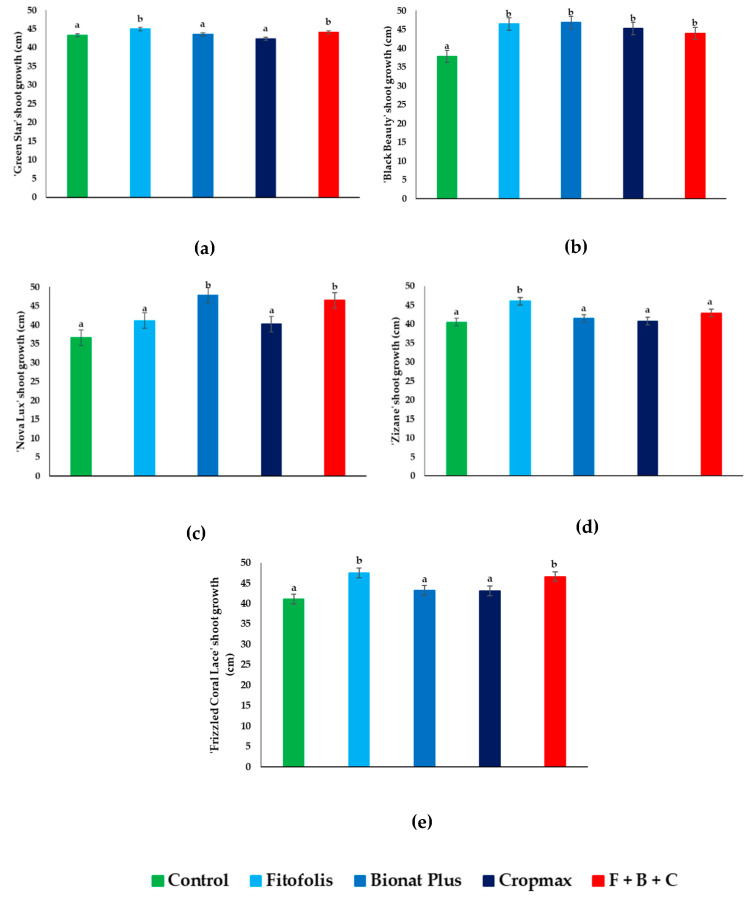
Effect of foliar fertilization on the shoot growth parameters in *Gladiolus* varieties: ‘Green Star’ (**a**), ‘Black Beauty’ (**b**), ‘Nova Lux’ (**c**), ‘Zizane’ (**d**), and ‘Frizzled Coral Lace’ (**e**). Plants shoot growth under control conditions, in the presence of the indicated foliar fertilization: Fitofolis, Bionat Plus, Cropmax and the mixture of Fitofolis–Bionat Plus–Cropmax (F + B + C). Shoot growth was measured in all plants just before starting the treatments (13 May), and before the harvesting of the inflorescences (9 August). Bars represent the means ± SE (*n* = 10). Different letters above the bars indicate significant differences between the treatments, according to Tukey test (α = 0.05).

Considering the shoot growth, almost all *Gladiolus* varieties showed small increases in shoot length (Figure 1). However, in the case of ‘Green Star’ (Figure 1a) the Fitofolis and the F + B + C (the mixture of Fitofolis, Bionat Plus and Cropmax) treatments influenced the shoot growth significantly compared to the control treatments. The effect of the treatment was evident, especially for the ‘Black Beauty’ gladioli, which showed an increase in growth in all treatments with respect to the controls, with ~10 cm (Figure 1b).

Regarding ‘Nova Lux’ (Figure 1c), only for the Bionat Plus and F + B + C treatments were significant differences reported; however, all treatments showed small increases (between 5–10 cm) compared to the control plants. An increase in mean shoot growth was also reported in ‘Zizane’ for the Fitofolis treatment (Figure 1d); for ‘Frizzled Coral Lace’ (Figure 1e), an increase of approximately 4 cm, was observed for the Fitofolis and F + B + C treatments.

Percentage increases in shoot growth of *Gladiolus* varieties influenced by the foliar fertilization were as follows: for ‘Green Star’, ‘Zizane’, and ‘Frizzled Coral Lace’ the highest percentage increase was recorded with Fitofolis (3.92%, 13.58%, and 15.54%) compared to the control. In contrast, the smallest percentage increases for the same three varieties were observed with Cropmax (0.02%, 0.61%, and 4.86%). The ‘Black Beauty’ reported the highest increase with Bionat Plus (23.81%) and the smallest percentage increase at F + B + C (16.27%). For ‘Nova Lux’, a 30.6% increase was found with Bionat Plus compared to only a 9.8% increase with Cropmax fertilization.

**Figure 2:**
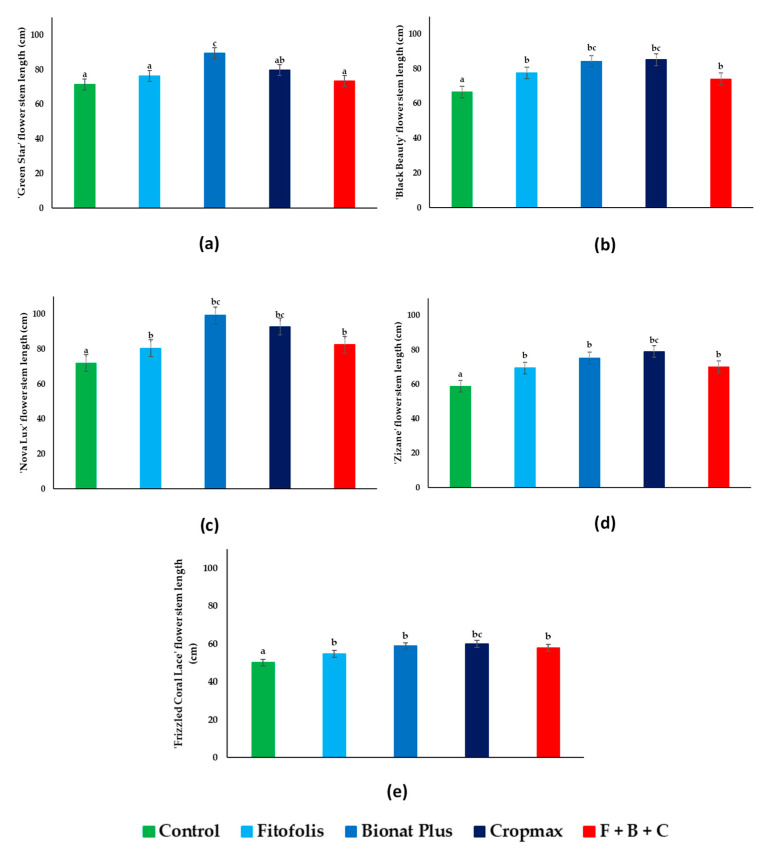
Effect of foliar fertilization on flower stem growth parameters in *Gladiolus* varieties: ‘Green Star’ (**a**), ‘Black Beauty’ (**b**), ‘Nova Lux’ (**c**), ‘Zizane’ (**d**), and ‘Frizzled Coral Lace’ (**e**). Plant flower stem growth shown under control conditions and in the presence of the indicated foliar fertilization: Fitofolis, Bionat Plus, Cropmax and the mixture of Fitofolis–Bionat Plus–Cropmax (F + B + C). Flower stem growth was measured in all plants just before starting the treatments (13 May), and before the harvesting of the inflorescences (9 August). Bars represent the means ± SE (*n* = 10). Different letters above the bars indicate significant differences between the treatments, according to Tukey test (α = 0.05).

The results of the present study indicated that a variety-specific response exists to foliar fertilization; in most cases the treatments significantly, positively influenced the flower stem growth (Figure 2) compared to the controls.

‘Green Star’ (Figure 2a) showed significant growth of the flower stem under the Bionat Plus fertilization, with a 19 cm increase; the other fertilization treatments influenced the flower stem growth, but at smaller percentages.

All types of foliar fertilization increased flower stem length for ‘Black Beauty’ (Figure 2b), compared to the control plants, in some cases by almost 20 cm. Regarding ‘Nova Lux’, ‘Zizane’ and ‘Frizzled Coral Lace’ *Gladiolus* (Figure 2c–e), similar results were found for all four fertilization treatments. For these three *Gladiolus* varieties, growth increase was between 5 and 30 cm depending on the treatment.

Comparing the stem growth between varieties in all treatments, the highest increase was observed in ‘Nova Lux’—almost 30 cm; the least growth was in ‘Frizzled Coral Lace’. This could be explained by the variety morphology.

When comparing the flower stem growth to the control, the greatest percentage increases in ‘Black Beauty’, ‘Frizzled Coral Lace’, and ‘Zizane’ were recorded with Cropmax (27.96%, 19.76%, and 34.35%), in ‘Nova Lux’ and ‘Green Star’ with Bionat Plus with 37.91% and 25.07%, respectively. The lowest percentage increases were observed with Fitofolis (‘Nova Lux’–11.82%, ‘Frizzled Coral Lace’–9.18%, and ‘Zizane’–18.02%) and F + B + C (‘Black Beauty’–11.27% and ‘Green Star’–2.66%) fertilizers.

### 2.2. Vase Durability

**Figure 3:**
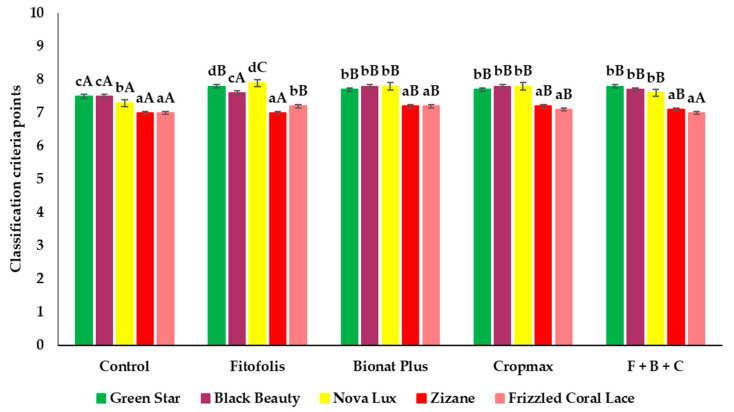
Effect of foliar fertilization on vase durability of *Gladiolus* varieties: ‘Green Star’, ‘Black Beauty’, ‘Nova Lux’, ‘Zizane’, and ‘Frizzled Coral Lace’. Vase durability of floral stems produced under control conditions and in the presence of the indicated foliar fertilization: Fitofolis, Bionat Plus, Cropmax and the mixture of Fitofolis–Bionat Plus–Cropmax (F + B + C). Bars represent the means ± SE (*n* = 5). Different lowercase letters above the bars indicate significant differences between the five varieties for each foliar fertilization, and different uppercase letters indicate significant differences between treatments, according to Tukey test (α = 0.05).

Under our experimental conditions, significant differences between the varieties and the treatments were observed in the vase durability of the *Gladiolus* (Figure 3). When comparing the varieties, it could be concluded that ‘Green Star’, ‘Black Beauty’ and ‘Nova Lux’ had the longest vase durability, whereas ‘Zizane’ and ‘Frizzled Coral Lace’ had shorter vase durability, with fewer points.

Foliar fertilizations influenced vase durability in a positive way, although with small differences. Almost all types of fertilization affected the gladioli durability, supporting the general conclusion of the individual experiments that all varieties responded to fertilization. The average vase durability was 7.45 days.

### 2.3. Daughter Corms Production

It was concluded that foliar fertilization had a positive effect on the increase in number of daughter corms production.

**Figure 4:**
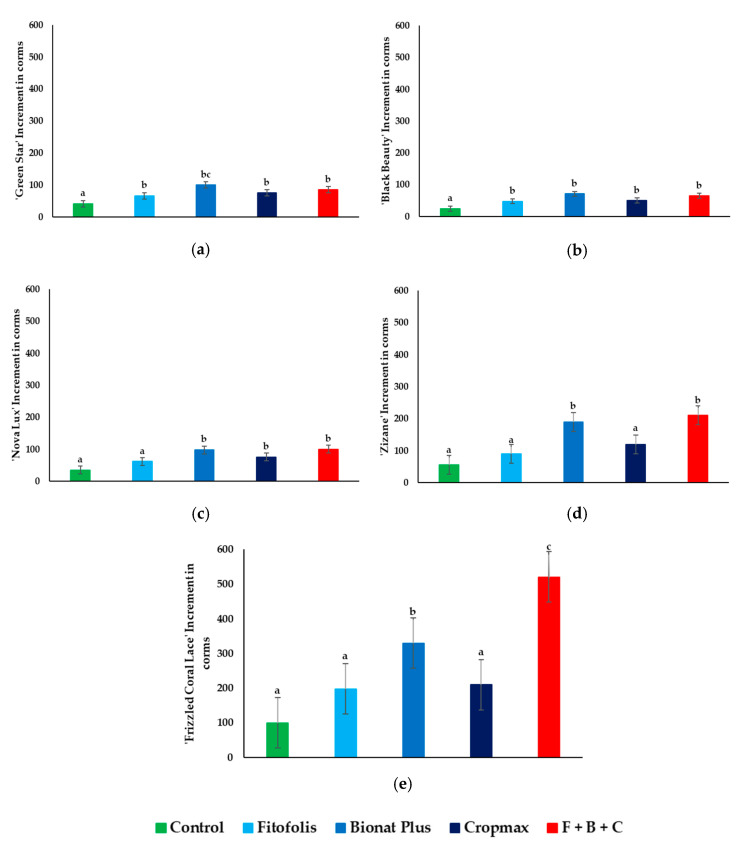
Effect of foliar fertilization on increment in daughter corms in *Gladiolus* varieties: ‘Green Star’ (**a**), ‘Black Beauty’ (**b**), ‘Nova Lux’ (**c**), ‘Zizane’ (**d**), and ‘Frizzled Coral Lace’ (**e**). Increase in daughter corms under control conditions and in the presence of the indicated foliar fertilization: Fitofolis, Bionat Plus, Cropmax and the mixture of Fitofolis–Bionat Plus–Cropmax (F + B + C). Bars represent the means ± SE (*n* = 10). Different letters above the bars indicate significant differences between the treatments, according to Tukey test (α = 0.05).

Under our experimental conditions ‘Green Star’ and ‘Black Beauty’ showed significant increases from all types of foliar fertilization, compared to the controls (Figure 4a,b).

For ‘Nova Lux’ (Figure 4c), there was no effect from the Fitofolis treatment. In contrast, the Bionat Plus, Cropmax and F + B + C treatments increased the corms production. Increases in the number of corms were also observed in ‘Zizane’ (Figure 4d) with the Bionat Plus and F + B + C treatments.

‘Frizzled Coral Lace’ (Figure 4e) showed a high increase from the F + B + C treatment, as daughter corms production was five times higher compared to the controls. This result could be influenced also by the variety: comparing the five different *Gladiolus* varieties, the greatest daughter corm production occurred in this variety.

In the cases of ‘Green Star’ (60.97%), ‘Black Beauty’ (92%), ‘Nova Lux’ (77.14%), and ‘Zizane’ (63.63%) the smallest percentage increase in relation to the controls were reported from Fitofolis. For ‘Frizzled Coral Lace’, the Cropmax fertilizer recorded the smallest increase in percentage with a 228.12% compared to control. The greatest increases were observed with Bionat Plus (‘Green Star’–143.9% and ‘Black Beauty’–184%) and the mixture of the three foliar fertilizers (‘Nova Lux’–185.71%, ‘Zizane’–281.81%, and ‘Frizzled Coral Lace’–714.06%).

## 3. Discussion

The results of this experiment show proper foliar fertilization can support and influence the growth, vase durability and daughter corms production of some *Gladiolus* varieties. Saima et al. [15] found that application of foliar spray affected flower production and it was the best method to getting maximum flower production in *Gladiolus*. Furthermore, it has a potential effect on the nutrient uptake and on the stimulation of growth parameters and flowering characteristics [5,16]. Foliar fertilization increases micronutrient uptake and physiological and biochemical indexes [17,18]. Many studies suggest that foliar fertilization may help to stimulate the uptake of soil applied fertilizers, which could provide a solution to salt accumulation in the soil [4].

Foliar fertilization was more effective and significantly enhanced the shoot and flower stem growth compared to the control plants. Similar to our study, some researchers reported that foliar fertilization promoted the flower stem growth to the maximum levels in gladioli, which could have a constructive role in the development of the flowers [15,19,20]. Similar findings have been described where the administration of foliar fertilization treatments influenced the *Calendula* inflorescence yield, but not the chlorophyll parameters, where no significant differences were observed between the treatments [21,22].

The data obtained clearly show that foliar fertilization can affect shoot growth in a positive way. Furthermore, the Fitofolis fertilizer obtained the best results compared to the control, which in some cases increased the growth up to 5 cm. The mixture of the three fertilizers (F + B + C) influenced shoot growth of gladioli in a positive way. In some varieties increases of 3 cm were shown compared to treatments with only Cropmax or Bionat Plus.

Like the shoot growth parameters, flower stem growth was influenced in a positive way by the foliar fertilization in all five varieties. Generally, the highest increases were observed in the plants fertilized with Bionat Plus, followed by Cropmax and Fitofolis. The mixture of foliar fertilizers in this case did not record as high an increase compared to the other three treatments. The macronutrients (N, P and K) are known to have effect on plant growth [23]. Nitrogen, phosphorus and potassium influenced the shoot growth and the flower stem length in a positive way. NPK used at an optimal dose can supplement sufficient nutrient uptake, which foster conditions for plants growth and development [24]. In some studies, it was also reported that B (boron) could increase–stimulate nutrient uptake, maintaining cell integrity and intensify respiration rate, which could promote growth and flower development [25,26,27].

Vase durability of *Gladiolus* is one of the most important considerations for consumers. Foliar fertilizer effects on vase durability have been reported on *Rosa* [28], *Lilium* [29], *Anthurium andreanum* [30] and *Gladiolus* [31,32]. In the present experiment, vase durability of the five gladioli varieties was improved compared to the control in almost all treatments. The longest vase durability was obtained under the Bionat Plus treatment, and the longest vase durability among the five varieties was observed for ‘Green Star’. *Gladiolus* fading or wilting are important signalling factors of senescence [33]. Calcium (Ca) has an important role in regulating the senescence in gladioli cut flowers [34]. Ca increases membrane stability and reduces the level of reactive oxygen species, which could delay senescence in *Gladiolus* cut flowers [35]. However, in a study conducted by Dhakal et al. [31] it was concluded that phosphorus could also improve vase durability of gladioli cut flowers.

Daughter corm production is an important part of the gladioli propagation; our study results clearly indicate foliar fertilization has an important role in this sequence of the cultivation. Previous reports have also shown an increase in daughter corms production under foliar fertilization treatments [16,36,37,38]. Under our experimental conditions the highest increase was recorded in ‘Frizzled Coral Lace’ compared to the other four *Gladiolus varieties*. The mixture foliar fertilization (F + B + C) improved the daughter corm production. This behaviour could be explained also with the variety characteristics, where previous results reported ‘Frizzled Coral Lace’ has a high yield of daughter corms. Daughter corm production could be influenced by nitrogen dose [39,40], and some studies have shown that daughter corm production is also influenced by applying a higher potassium dose [15,16,41].

## 4. Materials and Methods

### 4.1. Experimental Site and Plant Material

Open field experiments were conducted between April and September 2018 at the Sapientia Hungarian University of Transylvania, Târgu Mureș (46°31′17″ N 24°35′54″ E). The gladioli corms were obtained from Sieberz Garden Centre (Gödöllő, Hungary) and planted in five rows/block, each row containing 10 gladioli corms, with sizes of 12–14 cm in circumference. According to the soil analysis and of the analysis of its profile we can state that the type of soil at the experiment location is gley chernozem, carbonated in depth and clayish in the alluvial deposits (Epiaquic Hapludalfs) (Table 1).

**Figure 5:**
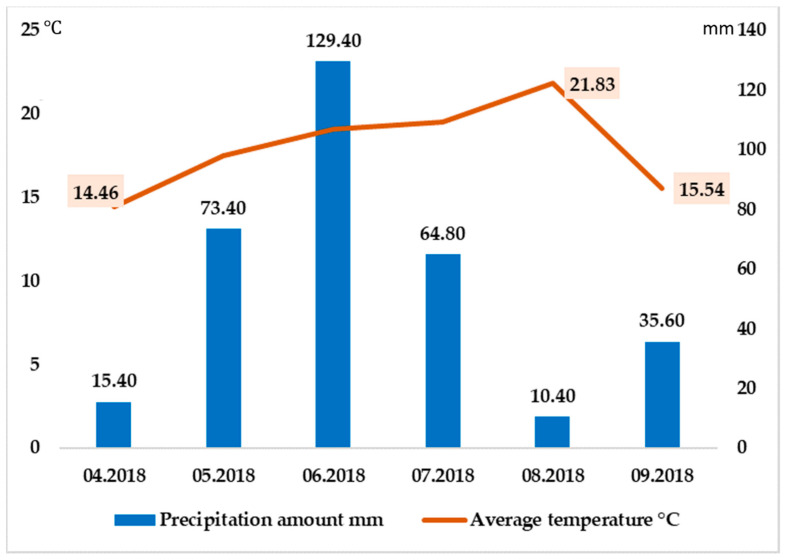
Meteorological conditions, precipitation and temperature during the field experiment (April–September 2018).

The average temperature during the experiment was 17.99 °C, the minimum was recorded in April (15.4 °C), and the maximum temperature in August (21.83 °C) (Figure 5). From Figure 5, it can be concluded that the average precipitation amount was 54.83 mm during the experimental months. The minimum precipitation was recorded in April at 15.40 mm, and the maximum in June was 129.40 mm. The precipitation and temperature data were collected using Delta–T devices WS–GP2 Automatic Weather Station (Delta-T Devices Ltd., Burwell, UK).

Morphological description of the five selected *Gladiolus* varieties:

- ‘Black Beauty’: flower colour dark burgundy; usually grows a flower stalk that is thin, straight, stiff; plant height 70–90 cm.
- ‘Green Star’: flower colour lime green; usually grows a flower stalk that is straight and vigorous; plant height 75–100 cm.
- ‘Nova Lux’: flower colour bright yellow; the flower stalk is thin but strong; plant height 80–110 cm.
- ‘Zizane’: flower colour combination of red and white; grows a straight, strong flower stalk; plant height 80–110 cm.
- ‘Frizzled Coral Lace’: flower colour coral pink; tends to grow more flower stems; plant height 60–100 cm.

*Gladiolus* corms were planted on 18 April 2018 with a row length of 25 cm and 15 cm between the plants. The plant growth was already observed at the end of the planting month.

### 4.2. Application of the Foliar Fertilization

On 13 May, two weeks after sprouting, first shoot measurements and the first foliar fertilization were made, according to the experimental design: A–Control, B–Fitofolis (Chemtech, Târgu Mureș, Romania), C–Bionat Plus (Panetone, Timișoara, Romania), D–Cropmax (Blondy, Târgu Mureș, Romania) and E–the mixture of the three foliar fertilizers (first it was fertilized with Fitofolis, the second fertilization was made with Bionat Plus, the third with Cropmax, and the last one with the mixture of the three fertilizers in 1:1:1 proportion) (Table 2).

The used foliar fertilizers content:

- Fitofolis: N–183 g/L; P–43 g/L; K–46 g/L; Fe–0.4 g/L; Cu–0.06 g/L; Mn–0.086 g/L; B–0.01 g/L; Zn–0.05 g/L; Mo–0.004 g/L.
- Bionat Plus: N–6.9%; P–0.003%; K–0.76%; Mg–0.47%; S–1.3%; Ca–0.05%; B–0.16%; Cu–0.35%; Fe–0.18%; Mn–0.06%; Zn–0.11%.
- Cropmax: N–0.2%; P–0.4%; N–0.02%; Fe–220 mg/L; Mg–550 mg/L; Zn–49 mg/L; Cu–35 mg/L; Mn–54 mg/L; B, Ca, Mo, Co, Ni–10 mg/L; auxin; cytokinin; gibberellin.

The application of the foliar fertilizers was done with a hand sprayer; for each product a 2% solution was prepared. To prevent the fertilization from getting into the wrong row, we placed a plastic film between the rows to protect them from the other treatments.

On 2 June the second foliar fertilization was applied; the only difference was that the gladioli on bed E were sprayed with the Bionat Plus. The third fertilization was made on 20 June, for the last (E) gladioli bed, Cropmax foliar fertilizer was applied.

The last fertilization was done on 5 July; E bed was fertilized with the mixture of the three foliar fertilizations (Fitofolis, Bionat Plus, Cropmax) in 1:1:1 proportions. The first flower buds appeared on 13 July. We measured the flower stem length of each plant. The last shoots measurements were made before harvesting the five *Gladiolus* varieties (9 August).

### 4.3. Vase Durability

When almost all gladioli started flowering (11 August) we randomly harvested five flowered stems, at a cut distance of 5 cm above the soil, from each treatment/*Gladiolus* variety. The vase durability was studied for seven days, and the gladioli cut flowers were kept under the same conditions: at room temperature, in clean-fresh water, and monitored and noted every three hours.

The classification criteria for the vase durability were determined as follows:

- Ten points if the flower buds are healthy, the lower inflorescence is fully open, the colour is bright and typical for the variety, the stem is flexible and straight, the leaves are brightly coloured.
- Nine points if the second and third flower buds have opened, the petal edges on the first are starting to wilt slightly, the stem is losing a little of its hold.
- Eight points when the fourth and fifth flower buds have opened, the petals of the lowest flower start to wilt.
- Seven points if lowest flower fully open, withered, leaves completely lose their glossy colour, stem loses elasticity.
- Two points when the uppermost buds in the inflorescence is opened, and the ones underneath are constantly wilting.

The above was used to determine the maximum value of vase durability.

### 4.4. Corms Propagation

At the end of August, all inflorescences were harvested from the plants; however, we left two leaves on each *Gladiolus* plant, thus contributing to the growth of the corms. In this way most of the photoassimilation is destined for the growth of the daughter corms. By the end of September, when the plants had stopped nutrient uptake and the remaining leaves withered, we gathered corms of five *Gladiolus* varieties. We dried them and cleaned the remaining soil of the corms.

### 4.5. Statistical Analysis

All data were tested for normality of errors and homogeneity of variance. As all data were normally distributed, ANOVA followed by Tukey test were used to compare variances. The significance of the differences between the treatments was tested by applying two-way ANOVA, at a confidence level if 95%. When the ANOVA null hypothesis was rejected, Tukey’s Post hoc test was carried out to establish the statistically significant differences at *p* < 0.05.

## 5. Conclusions

Gladioli growers strive to achieve the greatest possible stem length, vase durability and daughter corm production. The present study provides new experimental data on the responses of five *Gladiolus* varieties to foliar fertilization. For flower stem length and vase durability increase we recommend the use of Bionat Plus fertilizer, and for cut flowers the ‘Green star’ variety, which in our experiments had the best increases. The highest yield of daughter corm production was observed with the mixture of the three foliar fertilizations (F + B + C). These approaches/results will help to enhance the production of *Gladiolus*, with a smaller footprint on the degradation and salinization of the cultivated lands. Due to the frequent changes in cultivated *Gladiolus* varieties, we propose repeating this experimental design in a few years to examine the effect of foliar fertilization on new and requested varieties on the market.

## Figures and Tables

**Table 1:** Planted soil proprieties.

Depth (cm)	pH	P (ppm)	K (ppm)	Humus %	N %	Cohesion Coefficient	CaCO_3_ (ppm)
0–10	7.55	586	1850	7.29	0.348	48	1.33
10–20	7.48	520	1630	7.12	0.33	48.4	1.25
20–40	7.44	487	1550	6.22	0.304	48.8	

**Table 2:** Planting design: A: Control; B: Fitofolis; C: Bionat Plus; D: Cropmax; E: The mixture of Fitofolis, Bionat Plus and Cropmax (F + B + C).

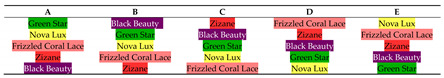

## Data Availability

Not applicable.
